# Cell stress molecules in the skeletal muscle of GNE myopathy

**DOI:** 10.1186/1471-2377-13-24

**Published:** 2013-03-12

**Authors:** Charlotte Fischer, Konstanze Kleinschnitz, Arne Wrede, Ingrid Muth, Niels Kruse, Ichizo Nishino, Jens Schmidt

**Affiliations:** 1 Department of Neurology, University Medical Center, Göttingen, Germany; 2 Department of Neuroimmunology, Institute for Multiple Sclerosis Research and Hertie Foundation, University Medical Center, Göttingen, Germany; 3 Department of Neuropathology, Prion and Dementia Research Unit, University Medical Center, Göttingen, Germany; 4 Department of Neuromuscular Research, National Institute of Neuroscience, Tokyo, Japan

**Keywords:** Cell stress, Nitric oxide, αB-crystallin, Myopathy, Skeletal muscle pathology

## Abstract

**Background:**

Mutations of the UDP-N-acetylglucosamine-2-epimerase/N-acetylmannosamine-kinase (GNE)-gene are causally related to GNE myopathy. Yet, underlying pathomechanisms of muscle fibre damage have remained elusive. In sporadic inclusion body myositis (sIBM), the pro-inflammatory cell-stress mediators αB-crystallin and inducible nitric oxide synthase (iNOS) are crucial markers of the disease pathology.

**Methods:**

10 muscle biopsies from GNE myopathy patients were analyzed for mRNA-expression of markers of cell-stress, inflammation and β-amyloid and compared to non-myopathic controls. Using double-labeling immunohistochemistry, serial sections of skeletal muscle biopsies were stained for amyloid precursor protein (APP), major histocompatibility complex (MHC)-I, αB-crystallin, neural cell adhesion molecule (NCAM), interleukin (IL)-1β, β-amyloid, iNOS, and phosphorylated neurofilament (P-neurofilament) as well as hematoxylin/eosin histochemistry. Corresponding areas of all biopsies with a total of 2,817 muscle fibres were quantitatively assessed for all markers.

**Results:**

mRNA-expression of APP, NCAM, iNOS, TNF-α and TGF-β was higher in GNE myopathy compared to controls, yet this was not statistically significant. The mRNA-expression of APP and αB-crystallin significantly correlated with the expression of several pro-inflammatory and cell-stress-associated markers as NCAM, IL-1β, TGF-β, CCL-3, and CCL4. By immunohistochemistry, αB-crystallin and iNOS were co-upregulated and the number of fibres positive for αB-crystallin, NCAM, MHC-I and iNOS significantly correlated with each other. A large fraction of fibres positive for αB-crystallin were double positive for iNOS and vice-versa. Moreover, several fibres with structural abnormalities were positive for αB-crystallin and iNOS. Notably, particularly normal appearing fibres displayed an overexpression of these molecules.

**Conclusions:**

The cell-stress molecules αB-crystallin and iNOS are overexpressed in GNE myopathy muscle and may identify early disease mechanisms. The data help to better understand the pathology of GNE myopathy.

## Background

GNE myopathy has previously been also termed quadriceps sparing myopathy, hereditary inclusion body myopathy, or distal myopathy with rimmed vacuoles. It is a slowly progressive myopathy that leads to wasting and weakness of distal and proximal muscles [1,2]. The disease mostly begins between the second and fourth decade of life and patients often loose ambulation after a disease course of 12 years or later [3]. Typical hallmarks of the muscle pathology include formation of vacuoles and tubulofilamentous inclusions by electron microscopy [4]. So far no treatment is available to effectively ameliorate the disease progression [5,6]. Various mutations have been identified in the UDP-N-acetyl-glucosamine 2-epimerase/N-acetylmannosamine kinase (GNE)-gene, a key enzyme of sialylation which is believed to be a major causative factor of the disease pathology [7,8]. Accordingly, mice with a knockout of the GNE gene develop a myopathy with features of GNE myopathy [9]. Moreover, there is evidence of hyposialylation in myoblasts from patients with GNE myopathy [10], yet the precise mechanism of how hyposialylation leads to damage of muscle fibers has yet remained elusive.

Inflammation is normally not present in GNE myopathy, although it is a typical hallmark in sporadic inclusion body myositis (sIBM), where it was recently shown that β-amyloid-associated molecules correlate with overexpression of inflammatory mediators [11]. Cell stress molecules such as αB-crystallin and nitric oxide (NO) have recently been identified in conjunction with the β-amyloid-associated pathology of sIBM [12,13]. NO can be produced in large amounts by inducible nitric oxide synthase (iNOS), which can be particularly upregulated under inflammatory conditions [14]. In view of previous evidence of a unique role of NO in GNE myopathy [15,16], we here searched for pro-inflammatory cell stress molecules in the muscle of GNE myopathy patients.

## Methods

### Patients and muscle biopsies

Diagnostic biopsies were used from skeletal muscle of 10 patients with GNE-mutations between 23–49 years of age, mean age of 33, six women and four men (Table 1). All samples were taken from the collection of the Department of Neuromuscular Research, National Center of Neurology and Psychiatry, National Institute of Neuroscience, Tokyo, Japan.

**Table 1:** List of patients with GNE myopathy

**Patient**	**Age**	**Gender**	**Mutation**	
1	>30	F	D176V	A630T
2	≤30	M	V572L	V572L
3	>30	F	V572L	V572L
4	≤30	M	D176V	V331A
5	≤30	F	V572L	V572L
6	>30	M	V572L	c.769 + 4A > G (exon4 skipping)
7	≤30	M	V572L	V572L
8	≤30	F	V572L	V572L
9	>30	F	D176V	V572L
10	>40	M	D176V	D176V

The age range at biopsy, gender and mutation details are indicated.

For the non-myopathic control group, nine muscle specimen were chosen from diagnostic muscle biopsies or orthopedic surgery at the University Medical Center, Göttingen, Germany: We used samples from six women and three men of 41–73 years of age, mean age of 52. The study was approved by the ethics committee of the Universities of Göttingen and Tokyo; an informed consent was not required.

### Extraction of RNA and quantitative PCR

Total RNA was extracted from muscle biopsies using a kit (RNeasy from Qiagen, Valencia, CA, USA), following the supplier’s instructions. The tissue was homogenized with a plastic tissue grinder and pestle (Kontes Glass Company, Vineland, NJ, USA) in 350 μl lysis buffer and the RNA was eluted in 30 μl water and stored at −80°C.

cDNA synthesis from 200 ng RNA was carried out using SuperScript II reverse transcriptase (Invitrogen, Darmstadt, Germany), following the supplier’s instructions. Originated cDNA was stored at −20°C and amplified in a 20 μl reaction volume with master mix for real-time PCR (Invitrogen) using 6-carboxy-fluorescein (FAM)-labelled probes and specific primers (Applied Biosystems, Carlsbad, CA, USA): Glyceraldehyde-3-phosphate dehydrogenase (GAPDH, s99999905_m1); APP (Hs00169098_m1), TGF-β1 (Hs00171257_m); IL-1β (Hs00174097_m1); CCL-3 (Hs00234142_m1); ubiquitin (Hs00430290_m1); CXCL-9 (Hs00171065_m1); αB-crystallin (Hs00157107_m1); NCAM (Hs00169851_m1); desmin (Hs00157258_m1); CCL-4 (Hs00605740_g1); IFN-γ (Hs00174143_m1); TNF-α (Hs00174128_m1); IL-6 (Hs00174131_m1) iNOS (Hs00167257_m1); MHC-I (custom design: forward 5’-TGG AGT GGC TCC GCA GAT AC-3’; reverse 5’-AGT GTG ATC TCC GCA GGG TAG A-3’). Reactions were performed in duplicates on a SDS 7500 Sequence Detection System (Applied Biosystems), following the standard cycle protocol and instructions given by the supplier. The resulting mRNA-expression was quantified using the Δc(t) method in relation to expression of GAPDH mRNA.

### Staining of muscle tissue

For immunohistochemistry, 5 μm frozen sections of all muscle biopsies were fixed either in PFA-methanol (4% PFA for 10 min, followed by methanol for 10 min) for iNOS and P-neurofilament (SMI-31) or in acetone at −20°C for 10 min for all other primary antibodies. Unspecific binding was reduced by 30 min incubation with 5% bovine serum albumin (BSA) and 3% goat serum (all from Jackson ImmunoResearch, West Grove, PA) in PBS. All primary and secondary antibodies were diluted in 1% BSA. Following primary antibodies were used at the respective concentration with an incubation time of one hour at room temperature unless stated otherwise: β-amyloid (mouse clone 6E10, Signet, Dedham, MA) at 10 μg/ml for 24 hours at 4°C; MHC class I (rat clone YTH 862.2 from Serotec, Oxford, UK) at 5 μg/ml; APP (rabbit polyclonal from Serotec, Oxford, UK) at 10 μg/ml; iNOS (rabbit polyclonal from Chemicon/Millipore, Billerica, MA) at 1/500 dilution; NCAM (mouse clone Eric-1 from Labvision/Neomarkers, Fremont, CA) at 2 μg/ml; αB-crystallin (rabbit polyclonal from Serotec) at 1/1000 dilution; P-neurofilament (mouse clone SMI-31 from Covance, Princeton, NJ, USA) at 0.5 μg/ml; IL-1β (rabbit polyclonal from Abcam, Cambridge, USA) at 1/100 dilution for 24 hours at 4°C.

Consecutive sections of all GNE myopathy patients were double-labelled for 1) APP and MHC-I; 2) NCAM and αB-crystallin; 3) IL-1β and β-amyloid; 4) iNOS and SMI-31, followed by a hematoxylin/eosin histochemistry. Secondary reagents were goat-derived Alexa 594 or Alexa 488 secondary antibodies. Nuclear counterstaining was performed by DAPI for 45 s at 1:50/000, followed by mounting in Fluoromount G (Southern Biotech, Alabama, USA). Digital photography was performed on a Zeiss Axiophot microscope (Zeiss, Göttingen, Germany), using appropriate filters for green (488 nm), red (594 nm) and blue (350 nm) fluorescence, a cooled CCD digital camera (Retiga 1300, Qimaging, Burnaby, BC, Canada) and the ImageProPlus software (MediaCybernetics, Bethesda, MD). Microphotographs of representative and corresponding areas of all serial sections were taken from each patient’s sample, yielding between 107 and 498 muscle fibres per patient. The expression of the respective markers was manually identified in a total of 2,817 fibres of all serial sections. A positive staining of individual fibers was judged by comparison with the signal intensities of the same biopsy, other samples from GNE-patients as well as normal control tissue.

### Statistics

For statistical analysis (*t*-test, Pearson correlation), *P < 0.05, **P < 0.01 and ***P < 0.001 were used as significant values and all significant outliers (Grubb’s test) were excluded prior to analysis (Graph Pad Prism V4, San Diego, CA, USA).

## Results

### mRNA-expression of disease relevant markers in GNE myopathy muscle compared to controls

**Figure 1:**
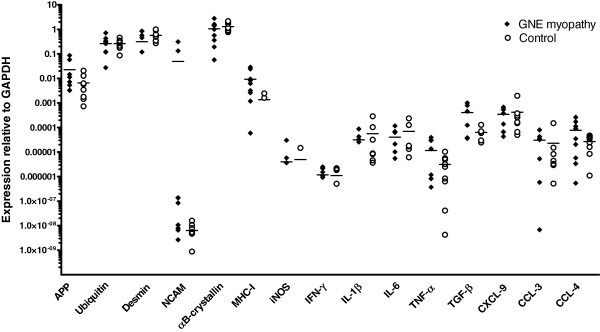
**mRNA-Expression of markers of inflammation, degeneration, regeneration and pro-inflammatory cell stress.** The mRNA-expression relative to GAPDH was quantified in all GNE myopathy samples compared to non-myopathic controls. The mean expression of APP, NCAM, MHC-I, TNF-α and TGF-β is higher in GNE myopathy compared to controls, but no statistical significance is reached.

Relevant markers of the β-amyloid-pathway (APP, UBB), muscle degeneration/regeneration (desmin/NCAM), cell-stress (αB-crystallin, MHC-I, iNOS) and inflammation (IFN-γ, IL-1β, IL-6, TNF-α, TGF-β, CXCL-9, CCL-3, CCL-4) as recently analyzed in sIBM and other inflammatory myopathies and non-inflammatory muscular dystrophy [11] were measured by quantitative PCR. All markers were detectable in GNE myopathy and the highest mean values of expression were noted for the degeneration-/cell stress associated markers αB-crystallin (1.052), desmin (0.315), ubiquitin (0.263), and APP (0.0226) (Figure 1). The mean expression of MHC-I remained at a slightly lower level (0.00925). The fibrosis-associated cytokine TGF-β (0.000407) and the chemokine CXCL-9 (0.000345) displayed a moderate expression level, whereas all other inflammatory mediators and NCAM (0.0494) were expressed at lower levels in most patients. Compared to non-myopathic control muscle, a higher mean expression was noted for APP, NCAM, MHC-I, TNF-α, and TGF-β, but no statistical significance was reached.

**Figure 2:**
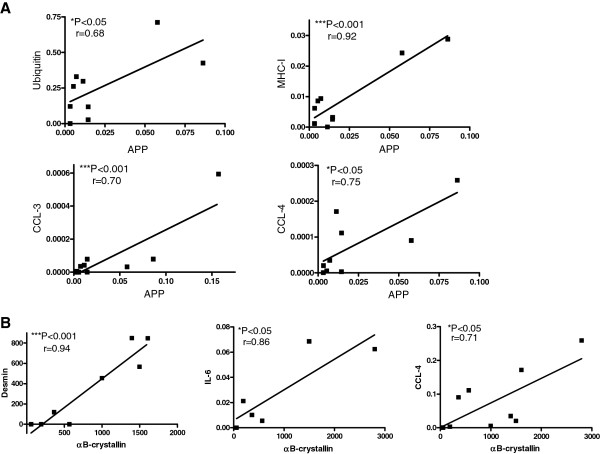
**Correlation analysis of mRNA- expression in GNE myopathy muscle.** The mRNA-expression of each marker from all GNE myopathy samples was correlated to other markers (data from Figure 1). **A**) The mRNA-expression of APP significantly correlates with that of UBB, MHC-I, CCL3 and CCL4. **B**) A significant correlation is observed between the cell stress marker αB-crystallin and desmin, IL-6 and CCL-4.

To address interrelationships between β-amyloid associated markers, cell-stress and inflammation, we performed a correlation analysis. As expected, the mRNA-expression of APP significantly correlated with the tagging molecule ubiquitin (Figure 2A). Moreover, APP was significantly associated with the cell-stress molecule MHC-I and the chemokines CCL-3 and CCL-4. Other markers remained without significant association with APP (not shown). The mRNA-expression of the heat shock protein αB-crystallin significantly correlated with desmin, a marker used to identify e.g. degeneration/regeneration, with the cytokine IL-6, and the chemokine CCL-4 (Figure 2B). Other associations with αB-crystallin did not reach statistical significance (not shown). Within the group of inflammatory mediators, the mRNA-expression of TGF-β significantly correlated with CCL-3 (r = 0.9786, P = 0.0007), CCL-4 (r = 0.9469, P = 0.0042) and TNF-α (r = 0.9080, P = 0.0123) (not shown). No obvious association between the most prevalent mutation V572L vs. other mutations and any of the pathologic markers was noted (not shown).

Collectively, these data demonstrate that markers of β-amyloid-associated pathology, cell-stress and inflammation are present in GNE myopathy muscle and significantly correlate with each other, albeit no significant overexpression could be observed.

### Protein expression of relevant markers of the disease

**Figure 3:**
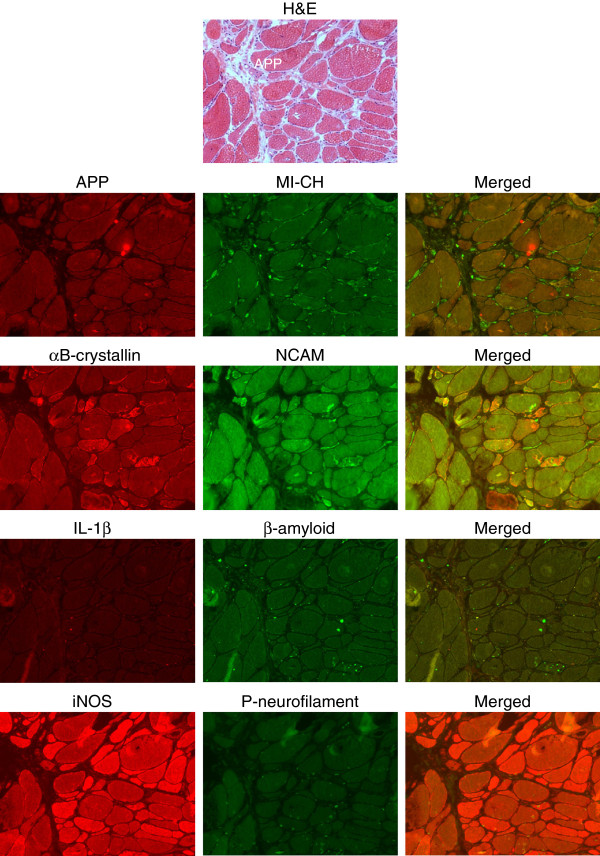
**Fluorescent immunohistochemical double-labelling of serial sections in GNE myopathy muscle biopsy specimen.** A representative GNE myopathy sample is shown with five consecutive sections that were stained for hematoxylin/eosin (first row); APP (red) and MHC-I (green) (second row); αB-crystallin (red) and NCAM (green) (middle row); IL-1β (red) and β-amyloid (green) (fourth row); iNOS (red) and P-neurofilament (green) (last row). The highest number of positive staining signal is noted for iNOS, followed by αB-crystallin and NCAM. All other markers are present only in very few fibers.

**Figure 4:**
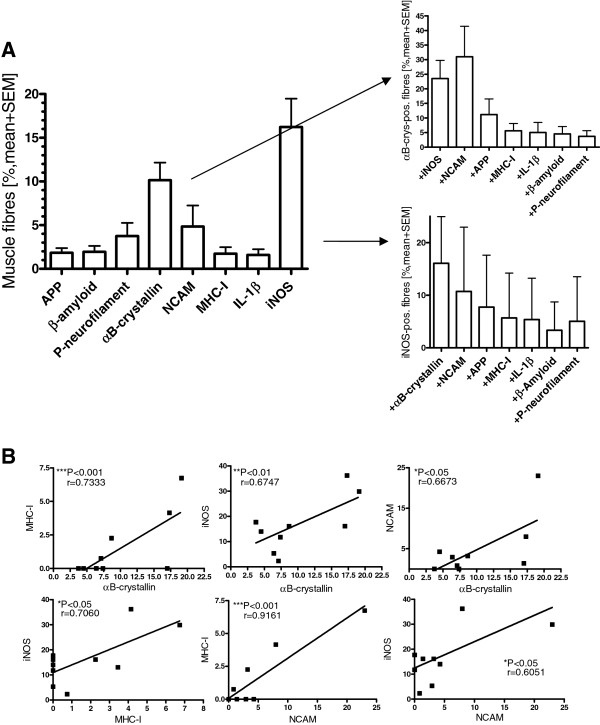
**Quantitative assessment of immunohistochemical staining in GNE myopathy.** The serial stainings as in Figure 3 were quantitatively assessed in all GNE myopathy samples. **A)** Percentages of all fibres positive for markers of degeneration, inflammation and cell stress in GNE myopathy (left panel, note the highest values for αB-crystallin and iNOS). Subtype analysis of fibres positive for αB-crystallin and iNOS (both panels on the right). Data presented as mean plus SEM. **B)** Correlation analysis of fibres positive for αB-crystallin, NCAM, MHC-I and iNOS by immunohistochemical staining demonstrates a significant association between these markers.

Protein expression was analyzed by serial staining of sections from biopsies of all GNE myopathy patients with immunohistochemical double labeling of 1) APP and MHC-I, 2) αB-crystallin and NCAM, 3) IL-1β and β-amyloid, and 4) iNOS and P-neurofilament. In a consecutive fifth section, histochemical staining for H&E was performed (Figure 3). By photomicroscopy and manual analysis, all entire cross sections of all 5 stainings from all GNE myopathy patients were quantified with an analysis of a total of 2,817 fibers. The two markers with the highest rate of positive fibers were αB-crystallin with 10.2 ± 2.0% (mean ± standard error) of all fibers and iNOS with 16.3 ± 3.2% of all fibers. NCAM was overexpressed in 4.8 ± 2.4% and P-neurofilament in 3.8 ± 1.5%, whereas APP, β-amyloid, MHC-I and IL-1β displayed a much lower expression (Figure 4A). A subtype analysis revealed that over a third of the fibers positive for αB-crystallin were double-positive for NCAM (33.8 ± 10.5%) and that 23.5 ± 6.2% were double-positive for iNOS. On the other hand, 15.9 ± 3% of the iNOS-positive fibers were double-positive for αB-crystallin and 10.7 ± 4.1% for NCAM. All other markers remained at a lower level. To further address the relevance of cell stress molecules, the correlation of their overexpression as reflected by immunohistochemistry was statistically analyzed. The number of fibers positive for the cell stress molecules iNOS and αB-crystallin highly significantly correlated with each other as well as with MHC-I and NCAM (Figure 4B). No significant association was observed for the other markers and no obvious association with the most prevalent mutation V572L vs. other mutations and any of the pathologic markers was noted (not shown).

Collectively, these data show that αB-crystallin and iNOS are the most prevalent cell stress markers in GNE myopathy muscle and that they significantly correlate and/or co-localize with other markers of inflammation and cell stress.

### Subtype analysis of fibers with or without structural abnormalities

**Figure 5:**
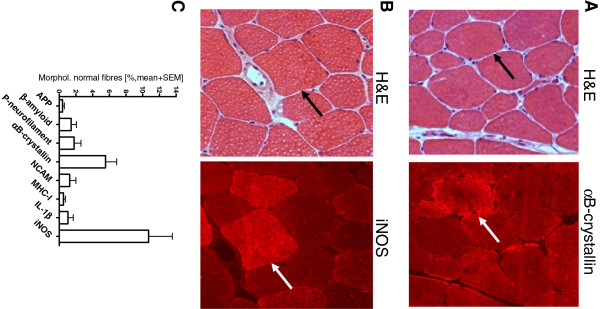
**Subtype analysis of GNE myopathy muscle fibres without structural abnormalities.** A subtype analysis of the serial stainings from Figure 3 was performed in all GNE myopathy samples. **A**) A representative fibre appearing normal by H&E staining (black arrow, left micrograph) displays overexpresssion of αB-crystallin per immunohistochemical staining (white arrow, right micrograph). **B**) A representative fibre appearing normal by H&E staining (black arrow, left micrograph) displays overexpresssion of iNOS per immunohistochemical staining (white arrow, right micrograph). **C**) Quantitative analysis of serial stainings in all GNE myopathy subjects reveals that αB-crystallin and iNOS are the most prevalent markers in normal appearing muscle fibers (data presented as mean plus SEM).

**Figure 6:**
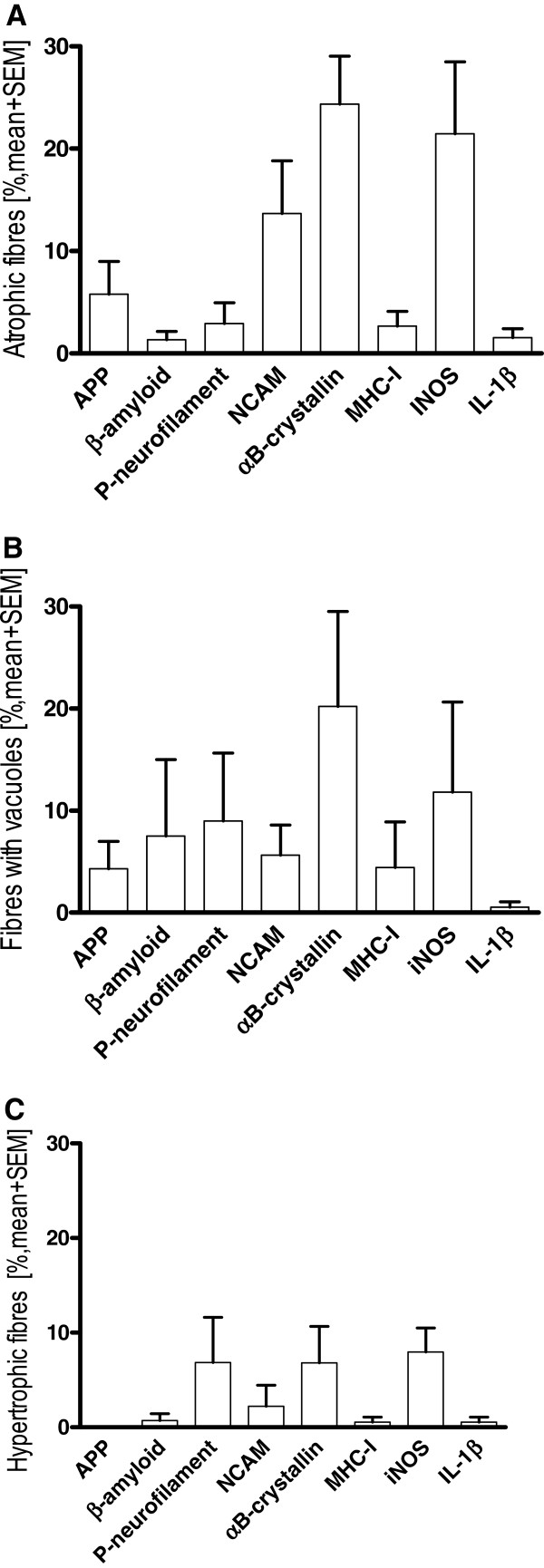
**Subtype analysis of morphologically altered fibres in GNE myopathy.** A subtype analysis of the serial stainings from Figure 3 was performed in all GNE myopathy samples. Quantification of immunohistochemical staining of markers of degeneration, inflammation and cell stress in atrophic fibres (**A**), fibres with vacuoles (**B**), and hypertrophic fibres (**C**).

To evaluate early cell stress mechanisms in GNE myopathy muscle, the upregulation of all relevant markers was analyzed in fibers that did not display any signs of morphological abnormalities (Figure 5). The only two markers that were noticeably overexpressed were αB-crystallin (5.5 ± 1.3%) and iNOS (10.7 ± 2.8%). 11.7% of the fibers positive for αB-crystallin were double positive for iNOS (data not shown). The other markers remained at a lower level. To further address interrelationships between cell-stress molecules, protein aggregation and muscle degeneration, a subtype analysis of fibers with signs of atrophy or hypertrophy was performed. In atrophic fibers, an overexpression was observed for αB-crystallin with 24.4 ± 4.7% positive fibers as well as for iNOS with 21.5 ± 7.0% of the fibers (Figure 6A), whereas all other markers remained at a lower level. In vacuolated fibers, αB-crystallin was present in a fraction of 20.2 ± 9.3%, followed by iNOS with 11.8 ± 8.8% (Figure 6B); all other markers were not noticeably upregulated. In hypertrophic fibers, the level of overexpression of iNOS (8 ± 2.5%), P-neurofilament (6.8 ± 4.8%) and αB-crystallin (6.8 ± 3.9%) were comparably elevated in contrast to all other markers that remained at a negligible level (Figure 6C).

Collectively, these data demonstrate that iNOS and αB-crystallin are present in normal appearing fibers and may precede subsequent morphological changes including atrophy, hypertrophy and vacuolar transformation.

## Discussion

We here demonstrate that αB-crystallin and iNOS are relevant cell stress markers in the muscle fibers of GNE myopathy patients. They are upregulated in normal appearing fibers as well as in those that had undergone atrophy, hypertrophy or vacuolar transformation. Pro-inflammatory cytokines and chemokines were expressed at levels comparable to control muscles, but some of these mediators were significantly associated with the markers for cell-stress or β-amyloid-associated degeneration. Collectively, our data suggest that a pro-inflammatory cell stress response with overexpression of αB-crystallin and iNOS is present in GNE myopathy muscle and precedes muscle degeneration with accumulation of β-amyloid.

The high frequency of an αB-crystallin signal in morphologically normal fibers is very similar to what has previously been demonstrated in sIBM [12,17] and suggests that an early underlying cell stress response is active in GNE myopathy. Since αB-crystallin has been shown to be important to protect from accumulation of unwanted proteins such as β-amyloid in muscle as well as in the brain [18-20], it is possible that muscle fibers upregulate αB-crystallin in order to downmodulate toxicity of APP and/or oligomers of β-amyloid, which may be present in muscle fibers even in absence of vacuoles or inclusion bodies [12]. This is in line with previous evidence that overexpression of APP in muscle cells led to an upregulation of αB-crystallin [21]. One fifth of the fibers with signs of an end-stage pathology with atrophic morphology and/or vacuoles were accompanied by an overexpression of αB-crystallin. This further substantiates that, once a cell stress response is instigated in the cells, a subsequent detrimental accumulation of unwanted proteins including β-amyloid will follow. Such an association of protein accumulation and αB-crystallin has previously been demonstrated in the skeletal muscle from patients with other myopathies [22].

Our data demonstrate that NO-related cell stress is present in muscle fibers of GNE myopathy patients, which is in line with previous reports [15,16]. This cell stress response does not appear to be specific to GNEmyopathy, but could be an underlying event with relevance to other myopathies: In sIBM, NO has recently been demonstrated to play an important pathogenic role as evidenced by an upregulation of the key NO-producing enzyme iNOS in muscle cells exposed to pro-inflammatory cytokines [13]. In other forms of myositis there is a similar production of NO upon overexpression of iNOS [23-25], which can be upregulated in response to inflammation. Since recent evidence has demonstrated that, in myositis, muscle fibers themselves can produce chemokines and cytokines [11], it is generally possible that a similar overexpression of inflammatory mediators also occurs in GNE-myopathy, even in absence of cellular infiltration. Yet other triggering events are likely to be more relevant in this disorder. It is even possible that NO-stress is a secondary event to accumulation of APP/β-amyloid, which would be supported by previous evidence of co-localization of oxidative stress related molecules with vacuoles and intracellular aggregation of proteins such as β-amyloid [16,26]. However, this would be in contrast to our present observation that iNOS is present in a substantial fraction of morphologically normal fibers and, thus, likely precedes vacuolar transformation and protein aggregation. On the other hand it is possible that vacuoles are already present at a different location within the same fiber.

A large fraction of morphologically normal fibers were double positive for αB-crystallin and iNOS, which further suggests that, similar to sIBM, an underlying cell stress response is operative in GNE myopathy muscle fibers. It is possible that similar triggers exist for an overexpression of αB-crystallin as well as for intrafiber-production of NO, but it cannot be excluded that one follows the other, e.g. that intracellular NO-toxicity leads to an overexpression of αB-crystallin and vice versa. The precise underlying conditions that lead to intracellular damage in GNE myopathy have yet remained elusive. A recent report has demonstrated that impaired sialylation leads to damage of the muscle tissue, which can be improved by sialic acid in a mouse model of the disease [27]. The same mechanism has also been shown to be operative in myoblasts from patients with GNE myopathy [7,10]. Thus, it is likely that hyposialylation precedes and is associated with a cell stress response around αB-crystallin and iNOS in GNE myopathy.

## Conclusions

Taken together, we here demonstrate that αB-crystallin and iNOS are early markers of a cell stress response in skeletal muscle fibers of GNE myopathy patients. Their presence and co-localization in normal appearing fibers suggests that they take precedence over accumulation of β-amyloid and formation of vacuoles. Despite absence of a cellular immune response, mRNA-expression of several inflammatory markers is present in GNE myopathy and some of them significantly correlated with αB-crystallin on the one hand and with APP on the other. Further evaluation of cell stress mechanisms in GNE myopathy may help to better understand the pathology of the disease and provide a useful tool for preclinical assessment of treatment strategies.

## Competing interests

The authors declare that they have no competing interests.

## Authors’ contributions

CF, KK, AW, IM, and NK conducted experiments. JS and CF wrote the manuscript. IN interpreted data and assisted in writing. JS designed the study. All authors approved the manuscript.

## Pre-publication history

The pre-publication history for this paper can be accessed here:

http://www.biomedcentral.com/1471-2377/13/24/prepub
